# A matched case-control study in Taiwan to evaluate potential risk factors for prostate cancer

**DOI:** 10.1038/s41598-023-31434-w

**Published:** 2023-03-16

**Authors:** Heng-Jui Chang, Yuan-Hung Pong, Chen-Yen Chiang, Po-Chien Huang, Ming-Hua Wang, Yu-Jiun Chan, Tzuo-Yun Lan

**Affiliations:** 1 Institute of Public Health, National Yang Ming Chiao Tung University, Taipei, Taiwan; 2 Department of Radiation Oncology, Wesing Surgery Hospital, Kaohsiung, Taiwan; 3 Department of Urology, Ten Chen Hospital, Taoyuan, Taiwan; 4 Division of Urology, Department of Surgery, Taoyuan General Hospital, Ministry of Health and Welfare, Taoyuan, Taiwan; 5 Department of Urology, Min Sheng General Hospital, Taoyuan, Taiwan; 6 Department of Radiation Oncology, Landseed International Hospital, Taoyuan, Taiwan; 7 Division of Microbiology, Department of Pathology and Laboratory Medicine, Taipei Veterans General Hospital, Taipei, Taiwan; 8 Center for Infection Control, Taipei Veterans General Hospital, Taipei, Taiwan; 9 Institute of Hospital and Healthcare Administration, National Yang Ming Chiao Tung University, No. 155, Sec. 2, Linong St., Beitou Dist., Taipei, 112304 Taiwan

**Keywords:** Cancer, Diseases, Oncology, Risk factors, Urology

## Abstract

The rising incidence rate of prostate cancer (PCa) worldwide has become a public health concern. PCa has a multifactorial etiology, and the link between human papillomavirus (HPV) and PCa has been widely investigated by numerous case–control studies. This age-matched, case–control study included 143 PCa patients and 135 benign prostatic hyperplasia (BPH) patients, with prostatic specimens testing negative for malignancy, as control. Study participants were recruited from four major hospitals in Taoyuan City, Taiwan, period 2018–2020, looking into HPV infection and other PCa risk factors, including dietary habits, family history, personal lifestyle, and sexual behavior. Multiple logistic regression analysis and forward stepwise selection analysis were conducted to identify potential risk factors for PCa. HPV DNA was found in 10 of the 143 PCa cases (7%) and 2 of the 135 BPH controls (1.5%) (OR = 6.02, 95% CI = 1.03–30.3, *p* = 0.046). This association was slightly significant, and furthermore, high risk HPV was not found to be associated with PCa. Higher body mass index (BMI) (OR = 1.15, 95% CI = 1.05–1.27, *p* = 0.003), more total meat consumption (OR = 2.74, 95% CI = 1.26–5.94, *p* = 0.011), exhibited association to PCa. However, PCa family history only presented a statistically significant difference by forward stepwise analysis (OR = 3.91, 95% CI = 1.17–13.12, *p* = 0.027). While much focus has been on the association between HPV and PCa, the results of this study indicate that more efforts should be directed towards investigating dietary habits, personal lifestyle and family history as factors for PCa. These results could serve as a basis for designing PCa prevention strategies.

## Introduction

In recent decades, with incidence rates of prostate cancer (PCa) on the rise worldwide, PCa has become an emerging public health issue in both developed and developing countries. PCa is now the second leading malignancy in men worldwide, with 1.4 million new cases and over 375,000 deaths in 2020 (GLOBOCAN)^1^. The relatively slow progression of PCa, along with developments in early detection and treatment, has led to a decreasing trend in mortality rates. USA cases diagnosed between 2001 and 2016 showed five-year survival rates of 98%^2^.

Comparing Taiwan and USA, epidemiological data of PCa for the two countries tell different stories. From 2000 to 2017, age-adjusted incidence rates in Taiwan doubled from 15.83 to 31.87 (per 100,000 males)^3,4^. In USA, age-adjusted incidence rates increased throughout the 1980s (from 105 to 170 per 100,000 men), peaked in the early 90 s (240 per 100,000), and has been in gradual decline since, to 107 per 100,000 (2017)^2,5,6^. Median age of diagnosis in USA is 66 years of age, whereas in Taiwan, it is 74. In USA, at time of initial diagnosis, only 13% had cancer in regional lymph nodes and 6% had developed distant metastasis. Comparable figures for Taiwan showed 18.35% of patients were stage III at initial diagnosis, while an astounding 33% were stage IV^7^. Bone metastases, lower urinary tract symptoms, asymptomatic pelvic lymphadenopathy, or asymptomatic liver/lung metastasis are commonly observed in such advanced stage PCa.

Catalona et al.^8^ published in 1991 showed that prostate specific antigen-based (PSA) screening could provide better PCa detection rates. Subsequent widespread adoption of PSA-screening in the early-90 s led to a spike in PCa incidence detection rates in USA. The improved early detection and ensuing treatment have been credited for the steady decline of incidence and mortality rates since the late-90 s. While many European countries have since enacted PSA-screening programs, Taiwan has yet to follow suit. The increasing rates of PCa, with relatively high rates of late-stage cases, may aggravate the financial burden of Taiwan’s National Health Insurance System, and should merit greater public awareness.

PCa is a multifactorial disease, and numerous epidemiologic studies have been conducted to survey various possible PCa risk factors^9–11^. Perdana et al. conducted a literature review study concluding that age, family history, insulin like-growth factors, dietary habit, lifestyle, environmental and occupational exposures were risk factors, while selected dietary products and supplementation may help in prevention^9^. Malik et al. had a multifactorial case-control study which demonstrated that age and smoking were risk factors, while fruits and vegetables could prevent PCa^10^. Pernar et al. provided descriptive epidemiology statistics and summarized that older age, family history, ethnicity, and genetics were strong risk factors^10^. Since the early-1950’s, sexually transmitted diseases (STD) have been linked as possible risk factors for PCa, with possible rationale being the repetitive inflammations caused by STD increasing cancerous growth and invasion of prostate cells. Initially, these studies were small, self-reported, or case-controlled with retrospective cohort, and generally focused on gonorrhea and syphilis. As new STD have been recognized, new resources for PCa research have been implemented to allow more prospective study designs, well-developed questionnaires, and serological/pathological assessments^12^. While several studies have explored relationships between STD and PCa, findings were inconsistent. Some studies have linked STD to cancer risks with increased odd ratios (OR): 1.2–1.5^13–15^, while other studies found no statistically significant difference^16,17^.

Human papillomavirus (HPV) is the most common viral STD worldwide. While cervical cancer was once the most common cause of cancer mortality for women in USA, advancements in screening (Pap and HPV) have allowed for early detection and treatment. Some studies have further investigated HPV in relation to PCa, with benign prostatic hyperplasia (BPH) patients as control group, and the results have been mixed. Studies that used seroprevalence of HPV antibodies in blood samples have mostly observed null results^18–22^, with plausible speculation being that serology substantiates presence of HPV infections in human body, but is unable to identify the definite source of infection. In studies where polymerase chain reaction (PCR), radical prostatectomy (RP), transrectal ultrasound (TRUS) biopsy, or transurethral resection of the prostate (TURP) were performed on prostate pathology samples, correlation of high risk HPV to PCa carcinogenesis or progression was disclosed, but overall findings were inconclusive^23–27^.

Results from several meta-analyses on HPV-PCa subjects have also been published. One such report analyzed 29 eligible studies (PCR or serologic), demonstrating that HPV slightly elevated risk of PCa (OR: 1.39, 95% CI: 1.12–2.06)^14^. Another meta-analysis of 25 eligible studies did not find increased PCa risk for HPV-16 (OR 1.09; 95% CI: 0.97–1.23) nor HPV-18 (OR 1.05; 95% CI: 0.89–1.24)^28^. However, results of secondary analysis indicated that only HPV-16 DNA prevalence was higher in PCa (OR: 1.54; 95% CI: 1.07–2.20). A similar study including 24 case-control studies involving 971 patients and 1,085 controls showed that the pooled estimate for OR was 2.27 (95% CI: 1.40–3.69)^29^. However, there were several limitations for these meta-analyses. Firstly, these individual case-control studies lacked power, as sample sizes were limited in number, and did not provide detailed calculation methods. Also, as is case with most case-control studies, possible information bias, selection bias, and difficulties in establishing temporal relationship, were factors to be considered. Secondly, some major confounders such as age, comorbidity, dietary habit, hygiene, occupational exposure, and sexual lifestyle were not adjusted for, leaving room for skepticism. Thirdly, previous studies were mostly conducted in Australia, Europe, and USA, lacking data and evidence from East Asian countries.

Aiming to overcome the previously observed shortcomings, we conducted a case-control study by including complete sample size calculation, age-matching between groups, and examining test–retest reliability. The intent of this case-control study was to identify potential PCa risk factors including detection of HPV infection, as well as dietary factors, family history, personal lifestyle, and sexual behavior.

## Methods

### Study design

This study utilized a prospective hospital-based, matched case-control design, at a 1:1 ratio. The case group consisted of 143 PCa patients, while the control group consisted of 135 BPH patients with prostatic biopsies testing negative for malignancy. Study participants were recruited between February 2018 and December 2020, from four major hospitals in Taoyuan City, Taiwan, upon approval from with each’s respective institutional review board (IRB). Patient age at enrollment was between 55 and 86. According to Taiwan’s Cancer Registry Annual Report for 2017, the median diagnostic age of PCa in Taiwan was 73^7^, whilst concerning incidence rates of PCa and BPH was relatively low for ages 55 years and younger. Patients aged 86 and over, were excluded due to perceived slow reaction times, poor physical performance statuses, and difficulties in comprehension of interview problems. Age was matched ± 5 years range between case and control groups. Exclusion criteria (assessed via questionnaire interview) included HPV-vaccinated as well as patients not initially diagnosed as PCa.

### Sample size calculation

A two-sided significance level (alpha) was set at 0.05, and power (% chance of detection) at 80%. Given that the sample size in most previous studies numbered only a few dozen, or lacked information on sample size calculation, these individual case-control studies were likely insufficiently powered. To overcome this limitation, we reviewed four related case-control articles which had clear HPV prevalence data in both groups: Singh et al.^23^: (39/95) 41% versus (11/55) 20%, Carozzi et al.^24^: (14/26) 53.8% versus (5/25) 0%, Leiros et al.^25^: (17/41) 41.5% versus (0/30) 0%, and Serth et al.^26^: (10/47) 21% versus (1/37) 3%. Three sample size calculators (http://osse.bii.a-star.edu.sg/calculation1.php, http://powerandsamplesize.com/, http://web1.sph.emory.edu/) were utilized to determine number of subjects for enrollment, with results of 74, 78, and 85. Patient drop-outs, refusals, or incomplete data, were forecasted to be 20–25%. As a result, 100 cases and 100 controls were determined to be the ideal sample size, and target recruitment was set at 120 for each group.

### Measure of exposures

We collected laboratory data from patients who agreed to provide prostate specimens at the urology departments of any of the four hospital sites. The main surgical procedures were radical prostatectomy (RP), transurethral resection of the prostate (TURP), and transrectal ultrasound biopsy (TRUS biopsy). RP was only performed in the PCa group. Informed consent forms were required. Potential PCa risk factors analyzed include detection of HPV infection, as well as dietary habits, family history, personal lifestyle, and sexual behavior.

A structuralized questionnaire was devised to identify for possible covariates, such as risk factors and exposures, during which 15 questionnaires and two experts were consulted. Based on an IRB request for patient privacy, the option to refuse answering for individual questions was allowed. The collected information included age, body mass index (BMI), diet, family history of cancer, occupational record, race, history of sexual activity and STDs, as well as use of tobacco, alcohol, and betelnut chew. The questionnaire was administered by qualified interviewers in-person. Only records of patients who provided both pathological specimens and completed the questionnaire were included in the data analysis.

### Measure of outcomes

Cases ascertained were those pathologically proven for PCa, aged 55 to 86. The majority of these cases had biopsies due to elevated-PSA and were suspected of PCa. A small number of cases were incidentally found to be malignant PCa during TURP for BPH symptoms. Selection of controls, or confirmed non-cases, were defined as BPH patients with clinically apparent lower urinary tract symptoms (LUTS), and were refractory to oral medicines, urethral catheterization, and received TURP from urologists. Another source of non-cases were patients with incidental PSA-elevation and highly suspected of PCa, whose TRUS biopsies detected no cancer cells.

HPV detection was performed in formalin-fixed, paraffin-embedded tissue (FFPE) samples by two commercial kits, Cobas 4800 HPV Test (Roche Molecular Systems) and DR HPV Genotyping IVD Kit (DR. Chip Biotech), at the Taipei Institute of Pathology. The Cobas 4800 HPV test individually detects HPV16 and HPV18, as well as pooled-detection of 12 other high risk HPV types (31, 33, 35, 39, 45, 51, 52, 56, 58, 59, 66, 68) by real time PCR. The DR HPV Genotyping IVD Kit (HPV-27) individually identifies 27 common HPV types, including 15 high risk (16, 18, 31, 33, 35, 39, 45, 51, 52, 56, 58, 59, 68, 73, 82) and 12 low risk types (6, 11, 53, 54, 61, 62, 66, 69, 70, 72, 81, 84).

The FFPE tissue specimen blocks were collected from the participating hospitals, deriving from different types of surgical procedures including RP, TRUS biopsy, and TURP. These FFPE blocks were sent to the Taipei Institute of Pathology where H&E staining slides had been prepared for pathologist review to confirm the diagnosis of malignant or benign disease. A minimum of three tissue sections, 5 um in thickness, per patient were collected for DNA extraction. DNA was extracted by using MagCore^®^ Genomic DNA Tissue kit (Cartridge Code 401, RBC Bioscience).

### Statistical analysis

Control and case groups were matched for age, and chi-square test was utilized to verify that there was no difference in distribution by age groups. Twenty patients from each group were selected for a second interview, within 30 days of each patient’s first interview, to validate the reliability of the questionnaire. Test–retest reliability coefficient was computed to measure the correlation between results from the first and second interview. Intraclass correlation coefficients (ICC) value of > 0.7 was deemed to be acceptable. As for the exposures or potential risk factors for PCa, independent t-test and chi-square test were adopted for univariate analysis of all variables and PCa status. If any individual variables reached statistical significance (*p* < 0.05), further multiple logistic regression analysis was conducted to determine the effect size of such single independent variables, in the presence of actual or potential confounding. Forward stepwise selection is a variable selection method, starting with a model that contains no variables (null model), then gradually adding the most significant variables one at a time, until it either reaches the stopping rule (criterion) or includes all of the variables into the model. The stopping rule (criterion) in this study is when *p* ≥ 0.05. The most significant variables are chosen from the result of simple logistic regression, including BMI, HPV detection, occupation exposure, etc.

### Reliability

The consistency of responses to the questionnaire was examined as follows: 20 cases and 20 controls, interviewed twice within 30 days, conducted by the same interviewer. Test–retest reliability was estimated by calculating the (CC) of the measured values at two separate time points. A higher CC between measured data denotes greater test–retest reliability, whereas Pearson’s *r*-value ≧ 0.7 indicates acceptable correlation. Uniformity of questionnaire interviews was enhanced by selecting one Principal Investigator (PI) and one assistant per hospital site, whereby the assistant was trained by the PI to conduct interview in similar manner.

### Ethical approval and consent to participate

This study was performed in line with the principles of the Declaration of Helsinki. We obtained IRB approval from National Yang Ming Chiao Tung University, Min Sheng General Hospital, Landseed International Hospital, Taoyuan General Hospital and Ten Chen Hospital. Informed consent was obtained from all individual participants included in the study.

## Results

### Basic characteristics

From Feb 2018 through Dec 2020, 278 subjects were recruited, with 143 in the case group and 135 in the control group (Table1). All subjects had filled out questionnaires and agreed to give pathology samples for laboratory examination. The mean age was 72.6 years (± 7.8 standard deviation [SD]) in case group, and 72.2 years (± 7.7 SD) in control group, with no difference (*p* = 0.161). Median age (with interquartile range) was 71.8 (11.2) and 71.3 (11.1) for case and control groups, respectively. Recruited patients were stratified into six subgroups (age 55 to 60, age 60.1 to 65, age 65.1 to 70, age 70.1 to 75, age 75.1 to 80, and age > 80 years). The chi-square test showed that the age distribution between groups were not different, with p value of 0.826, substantiating the age-matching method. Mean subject height was 164.8 cm (± 5.9 SD) for case group, and 166.7 cm (± 6.0 SD) for control group, with significant difference (*p* = 0.007). Mean subject weight was 68.2 kg (± 9.4 SD) in case group and 66.2 kg (± 10.1 SD) in control group, with no significant difference (*p* = 0.091). The mean BMI was 25.7 kg/m^2^ (± 3.2 SD) in cases and 23.8 kg/m^2^ (± 3.3 SD) in controls, with significant difference (*p* < 0.001). In terms of occupational exposure, i.e., chemical solutions/materials, plastic materials, metal dust, machinery, electroplating, printing ink, dyes, paints, radiation, and electromagnetic field, subjects in PCa group had a significantly higher proportion than patients in BPH group (33.8% vs 19.3%, *p* = 0.006). BPH was more remarkable in control group than in case group, 94.8% versus 79.0% (*p* < 0.001), as selection for control group was largely from BPH patients with clinically apparent LUTS, as well as patients with incidentally determined PSA elevation.

**Table 1:** Socio-demographic, occupational, personal lifestyles and clinical characteristics.

Characteristic	Stratification	A-Case (N = 143)		B-Control (N = 135)		*P* value
		N	%	N	%	
Age (Mean ± SD)		72.6 ± 7.8		72.2 ± 7.7		0.161
Age (Median&IQR)		71.8 (11.2)		71.3 (11.1)		
Height (Mean ± SD) (cm)		164.8 ± 5.9		166.7 ± 6.0		0.007*
Weight (Mean ± SD) (kg)		68.2 ± 9.4		66.2 ± 10.1		0.091
BMI (Mean ± SD) **(**kg/m^2^)		25.7 ± 3.2		23.8 ± 3.3		< 0.001*
Occupational	Ever	48	33.8	26	19.3	0.006*
exposure	Never	94	66.2	109	80.7	
HPV detection	Positive	10	7.0	2	1.5	0.024*
	Negative	133	93.0	133	98.5	
HPV type		18(2), 52(1), 53(3), 62(1), non-16/18(3)		52(2)		
BPH	No	30	21.0	7	5.2	< 0.001*
	Yes	113	79.0	127	94.8	
Meat consumption	< 1 serving/meal	24	18.2	68	50.4	< 0.001*
	≧ 1 serving/meal	108	81.8	67	49.6	
Main type of meat	White meat/seafood	22	16.7	39	30.7	0.008*
	Red meat	110	83.3	88	69.3	
Red meat percentage	< 25%	30	22.7	58	45.7	< 0.001*
	25–50%	42	31.8	41	32.3	
	> 50%	60	45.5	28	22.0	
Frequency of dairy product	Never	33	25.0	46	34.1	0.052
	1–3 times per week	57	43.2	65	48.1	
	≧ 4 times per week	17	12.9	11	8.1	
	Almost everyday	25	18.9	13	9.6	
Daily vegetable consumption	< 1 meal	15	11.4	4	3.0	0.010*
	1–2 meals	68	51.5	64	47.4	
	≧ 3 meals	49	37.1	67	49.6	
Daily fruit Consumption	< 1 meal	42	31.8	26	19.3	0.027*
	1–2 meals	76	57.6	84	62.2	
	≧ 3 meals	14	10.6	25	18.5	
Soybean product consumption	No	72	54.5	56	41.5	0.006*
	1–2 types	38	28.2	34	25.2	
	3–4 types	22	16.7	45	33.3	
Vegetarian	No	143	100	127	94.1	0.003*
	Yes	0	0	8	5.9	
Circumcision	Never	112	80.0	122	90.4	0.016*
	Ever	28	20.0	13	9.6	
IPSS (mean ± sd)		14.1 ± 7.8		17.3 ± 7.3		< 0.001*

*SD* standard deviation, *BMI* body mass index, *Univ.* university, *FT* full-time, *PT* part-time, *RET* retired, *UE* unemployed, *IQR* interquartile range.

*P < 0.05; The number in parenthesis ( ) in HPV type denotes case number.

We detected 10 out of 143 PCa patients as well as 2 out of 135 BPH patients to be HPV positive, with positive detection rate of 7% versus 1.5% (*p* = 0.024), which demonstrates greater HPV correlation in the PCa group. Revealed HPV types were 18 (2), 52 (1), 53 (3), 62 (1), non-16/18 (3) in case group, while type 52 (2) was only noted in control group, with number in parenthesis denoting number of HPV cases. HPV types 18, 52, 58, 62 were high risk while non-16/18 was low risk. However, the association between total HPV detection and PCa was slightly significant in further logistic regression analysis. If low risk types were not included, then high risk HPV detection was not associated with PCa.

Other non-significant variables including education level of subjects, marital status, working status, as well as paternal and maternal tongue were shown in supplements (Table S1).

### Medical history

History of radiation therapy (RT), dietary supplement, and medication history was illustrated in Table S2. RT history was defined as any radiation treatment for any cancer type other than PCa prior to recruitment, where no difference was found between the groups. Popular dietary supplements among adults in Taiwan include calcium, vitamin B, vitamin C, vitamin complex, zinc, selenium, green tea powder, isoflavone, royal jelly, phytoestrogen essential, iron, omega-3 fatty acid, vitamin D, glucosamine, probiotic, lutein, chicken essence, amongst others. We observed no difference in dietary supplement intake between two groups. Numerous patients had history of taking medications, and there was no difference observed in drug history. Chronic inflammation was proved to induce carcinogenesis in many ways, and PCa was also hypothesized to be correlated with pelvic surgery, pelvic disease, or other status related with chronic inflammation. However, we observed no significant difference in urinary calculi (*p* = 0.616), urinary tract infection (*p* = 0.174), bladder disease (*p* = 0.833), pelvic trauma (*p* = 0.859), pelvic surgery (*p* = 0.709), autoimmune disease (*p* = 0.486), pelvic cancer (*p* = 0.531), and extrapelvic cancer (*p* = 0.061) between the groups (Table S3).

### Personal lifestyles

Differences in personal lifestyles, especially in dietary habit, were noted (Table 1). Meat consumption ≧ 1 serving per meal was 81.8% in the case group and 49.6% in the control group, (*p* < 0.001). Meat consumption was categorized as white meat, seafood, and red meat. The majority of subjects in both groups ate red meat primarily, but red meat consumption was significantly higher in case group than control group (83.35% vs 69.3%, *p* = 0.008). We further explored meat consumption by questioning subjects as to percentage of total meat consumed being red meat, as stratified into three groups: less than 25%, between 25 and 50%, and greater than 50%. Case group was 22.7%, 31.8%, and 45.5%, respectively, whereas control group was 45.7%, 32.3%, and 22.0% (*p* < 0.001). Case group also reported greater update of dairy products, although not statistically significant (*p* = 0.052). Case group also consumed fewer vegetables (*p* = 0.01), fewer fruits (*p* = 0.027), and fewer soybean products than the control group. An interesting observation was that all vegetarian subjects belonged to the control group, while none were found in the case group (*p* < 0.003), in line with the possibility that vegetarian diets are a preventative factor for PCa.

Other non-significant variables were shown in Table S4. Consumption of tea drink, alcohol and tobacco were similar in both groups. Endemic to Taiwan and other parts of East Asia is betelnut chewing, which has strong association with oral cancer and other systemic effects. However, we did not observe the correlation between betelnut habit and PCa in this study (*p* = 0.173).

### Reproductive history and family history

Family history of PCa is a complex mix of genetic and environmental factors, and it is an important parameter that can be assessed in clinical practice through basic questionnaires^30^. This study also explored family history of PCa via questionnaire, and results revealed positive correlation between PCa and family history of PCa, with 11.4% in case group and 3.7% in control group, with family history of PCa (*p* = 0.016) (Table 2). However, further investigation (Table S5) into which family member had diagnosis of PCa, such as grandfather, granduncle, father, uncle, brother, cousin, son, or grandson, did not show significance perhaps due to inadequate sample size. International Prostate Symptom Score (IPSS) is a tool with 7 questions related to different urinary symptoms (incomplete emptying, frequency, intermittency, urgency, weak stream, and straining), which allows urologists to better understand the severity of BPH. IPSS can score from 0 to 35. In Table 1, the mean IPSS was 14.1 (± 7.8 SD) in case group and 17.3 (± 7.3 SD) in control group with difference (*p* < 0.001), and score summation 8–19 was deemed as moderate BPH. Circumcision before first sexual intercourse was associated with a slightly lower reduction in the relative risk of PCa in some studies^31^, but inconclusive. Our study showed contradictory results, with more patients in case group than control having received circumcision (20.0% vs 9.6%, *p* = 0.016) (Table 1). However, more than 90% of patients in our study had circumcision after adulthood, but not before first sexual intercourse.

**Table 2:** Sexual characteristics, history of other sexually transmitted infections.

Characteristic	Stratification	A-Case (N = 143)		B-Control (N = 135)		*P* value
		n	%	n	%	
Family history of PCa	No/Unknown	124	88.6	130	96.3	0.016*
	Yes	16	11.4	5	3.7	
Lifetime sexual partner	≦ 1 person	46	32.2	65	48.1	0.007*
	2–3 persons	31	21.7	31	23.0	
	≧ 4 persons	66	46.2	39	28.9	
STD history	Never/unwilling to tell	114	80.3	124	91.9	0.003*
	1STD	26	18.3	7	5.2	
	≧ 2 STDs	2	1.4	4	3.0	
Lifetime spouse	1 person	128	89.5	130	96.3	0.029*
	≧ 2 persons	15	10.5	5	3.7	
Lifetime sexual partner	1 person	127	88.8	129	95.6	0.037*
	≧ 2persons	16	11.2	6	4.4	

*P < 0.05.

The two groups were not different in regard to history of androgen replacement therapy (for hypogonadism) or anti-alopecia agent (such as finasteride) (Table S6). Vasectomy was linked to a slight increase in long-term risk of PCa in some studies^32^, but this issue remains controversial. In our study, 3.6% of case group and 7.4% of control group have received vasectomies, without significant difference (*p* = 0.161). The condition of genital beading and tattoo over lower abdomen or genital organ, and the experience of HPV vaccination was not different between groups. International Index of Erectile Function (IIEF-5), consisting of 5 questions, has evolved to be the standard for evaluating the severity of erectile dysfunction (ED). The 5 domains are erectile function, orgasmic function, sexual desire, intercourse satisfaction, and overall satisfaction, with score ranging from 5 to 25. IIEF-5 was 8.1 (± 9.5 SD) in PCa group and 8.3 (± 9.5 SD) in BPH group, without difference (*p* = 0.842), and score summation 8–11 was defined as moderate ED.

### Sexual behavior and STD history

PCa patients had more lifetime sexual partners than BPH patients (*p* = 0.007), with 46.2% of PCa patients having had ≧ 4 partners throughout their life, while figure was only 28.9% in BPH patients (Table 2). Meanwhile, the proportion of ≦ 1 partner was 32.2% versus 48.1%. STDs have been linked as potential risk factors to PCa, and in our study, 19.7% in case group had one or more STD, compared to 8.2% in control group, *p* = 0.003. Table S7 showed non-significant variables between case and control group in sexual orientation (heterosexual 97.0% vs 99.3%, *p* = 0.372), number of marriages (≧ twice 9.1% vs 6.7%, *p* = 0.454), number of children (≧ 2 children 89.5% vs 87.4%, *p* = 0.583), and sexual partners in recent one year (*p* = 0.709). As to the prevalence of condom use, control group had greater proportion of using condom than case group (56.3% vs 43.7%), but without significance (*p* = 0.094). The case group showed greater patronage of prostitutions throughout their life, with 32.9% having ≧ 3 prostitutes vs 20.7% in control group, although not statistically significant (*p* = 0.057).

We also questioned subjects regarding sexual behavior and reproductive status of their wives (Tables 2 and S88). We found that the case group had more spouses in lifetime (*p* = 0.029) and sexual partners (*p* = 0.037). History of STD in spouses (*p* = 1.000) and cancer history (*p* = 0.904) was not different between two groups. HPV vaccines were launched in Taiwan circa 2006, and generally applied to adolescents and young adults, so almost none of the wives in either group had received vaccination for HPV (*p* = 0.235).

### Logistic regression and other analysis

Results of simple and multiple logistic regression (LR) of risk factors associated with PCa were shown (Table 3). PCa risk was found to be significantly associated with higher BMI (*p* < 0.001 and *p* = 0.003), HPV positivity (*p* = 0.040 and *p* = 0.046), and meat consumption (*p* < 0.001 and *p* = 0.011) in both simple and multiple LR. IPSS summation score of PCa was lower than that of BPH, which was due to essence and definition of controls selection. PCa-associated risk factors, in only simple LR but not in multiple LR, were occupational exposure (*p* < 0.001), eating red meat as main protein (*p* = 0.009), higher red meat consumption (*p* < 0.001), PCa family history (*p* = 0.022), circumcision history (*p* = 0.018), ≧ 4 lifetime sexual partners (*p* = 0.002), suffering 1 type of STD (*p* = 0.002), wife’s lifetime spouse ≧ 2 (*p* = 0.036), and wife’s lifetime sexual partners ≧ 2 (*p* = 0.044). Potential protective factors for PCa, in only simple LR but not in multiple LR, were daily consumption of more vegetables (*p* = 0.006), fruits (*p* = 0.011), and soybean products (*p* = 0.002). Further forward stepwise LR (Table 4) confirmed that risk factors for PCa were meat consumption ≧ 1serv/meal (OR = 3.87, 95% CI = 2.07–7.25, *p* < 0.001), BMI (OR = 1.15, 95% CI = 1.05–1.25, *p* = 0.002), positive HPV infection (OR = 6.67, 95% CI = 1.28–34.74, *p* = 0.024), and PCa family history (OR = 3.91, 95% CI = 1.17–13.12, *p* = 0.027).

**Table 3:** Logistic regression analysis of risk factors associated with PCa.

Variable	Simple logistic regression			Multiple logistic regression		
	Crude OR	95%CI	*P* value	Adj-OR	95%CI	*P* value
BMI	1.21	(1.11, 1.31)	< 0.001*	1.15	(1.05, 1.27)	0.003*
HPV result						
Negative(Ref.)	1.00			1.00		
Positive	5.00	(1.08, 23.26)	0.040*	6.02	(1.03, 30.30)	0.046*
Occupation exposure						
No(Ref.)	1.00			1.00		
Yes	2.14	(1.23, 3.72)	< 0.001*	1.58	(0.78, 3.17)	0.203
Meat consumption						
< 1 serv/meal (Ref.)	1.00			1.00		
≧ 1 serv/meal	4.57	(2.62, 7.97)	< 0.001*	2.74	(1.26, 5.94)	0.011*
Main type of meat						
WM/SF (Ref.)	1.00			1.00		
Red meat	2.22	(1.23, 4.01)	0.009*	0.73	(0.28, 1.89)	0.517
Red meat percentage						
< 25% (Ref.)	1.00			1.00		
25–50%	1.98	(1.07, 3.67)	0.030*	2.10	(0.83, 5.29)	0.115
> 50%	4.14	(2.21, 7.77)	< 0.001*	2.54	(0.91, 7.09)	0.075
Daily vegetable						
< 1 meal (Ref.)	1.00			1.00		
1–2 meals	0.28	(0.09, 0.90)	0.032*	0.26	(0.06, 1.16)	0.078
≧ 3 meals	0.20	(0.06, 0.62)	0.006*	0.33	(0.07, 1.52)	0.155
Daily fruit						
< 1 meal (Ref.)	1.00			1.00		
1–2 meals	0.56	(0.31, 1.00)	0.050	0.63	(0.30, 1.33)	0.226
≧ 3 meals	0.35	(1.53, 0.79)	0.011*	0.62	(0.21, 1.81)	0.383
Soybean product						
Never	1.00			1.00		
1–2 products	0.87	(0.49, 1.55)	0.636	0.82	(0.39, 1.72)	0.591
3–4 products	0.38	(0.21, 0.71)	0.002*	0.57	(0.26, 1.23)	0.150
Vegetarian						
No (Ref.)	1.00			1.00		
Yes	0.00	0.00	0.999	0.00	0.00	1.000
Circumcision						
No (Ref.)	1.00			1.00		
Yes	2.35	(1.16, 4.75)	0.018*	1.85	(0.78, 4.35)	0.161
IPSS	0.95	(0.91, 0.98)	0.001*	0.95	(0.92, 0.99)	0.020*
PCa family history						
No (Ref.)	1.00			1.00		
Yes	3.36	(1.19, 9.43)	0.022*	3.41	(0.89, 13.09)	0.074
Lifetime sexual Partner						
≦ 1 person (Ref.)	1.00			1.00		
2–3 persons	1.41	(0.76, 2.64)	0.278	0.85	(0.39, 1.84)	0.682
≧ 4 persons	2.39	(1.38, 4.13)	0.002*	0.79	(0.36, 1.74)	0.553
STD						
Never (Ref.)	1.00			1.00		
1 type	4.04	(1.69, 9.67)	0.002*	2.09	(0.70, 6.25)	0.187
≧ 2 types	0.54	(0.10, 3.03)	0.487	0.21	(0.02, 2.24)	0.195
Wife’s lifetime spouse						
1 person (Ref.)	1.00			1.00		
≧ 2 persons	3.05	(1.08, 8.63)	0.036*	5.92	(0.27, 128.80)	0.768
Wife’s lifetime sexual partner						
1 person (Ref.)	1.00			1.00		
≧ 2 persons	2.71	(1.03, 7.14)	0.044*	0.656	(0.04, 10.52)	0.768

*P < 0.05.

**Table 4:** Forward stepwise logistic regression of risk factors for PCa.

Variable	β	SE	Wald	OR	95%CI	*P* value
Meat consumption						
< 1 serv/meal (Ref.)				1.00		
≧ 1serv/meal	1.35	0.32	17.84	3.87	(2.07, 7.25)	< 0.001*
BMI	0.14	0.04	9.39	1.15	(1.05, 1.25)	0.002*
IPSS	− 0.05	0.02	6.12	0.96	(0.92, 0.99)	0.013*
HPV detection						
Negative (Ref.)				1.00		
Positive	1.90	0.84	5.08	6.67	(1.28, 34.74)	0.024*
PCa family history						
No (Ref.)				1.00		
Yes	1.37	0.62	4.89	3.91	(1.17, 13.12)	0.027*

*P < 0.05.

Test–retest reliability of the questionnaire was assessed by calculating the correlation coefficient (CC), and all the information was collected in supplement (Table S9). Twenty cases, 20 controls, and a sum of 40 patients were evaluated. Among the 55 evaluable questions, all Pearson correlation coefficients (r) in 40 patients were ≧ 0.7. In the case group, (r) for all 20 subjects were ≧ 0.7, while in the control group, 96.4% (53/55) were ≧ 0.7. Overall result proved acceptable correlation.

## Discussion

To our knowledge, this is the first age-matched case-control study in East Asia to look at HPV infection, dietary habit, and other potential risk factors for PCa, using pathology specimens with questionnaires, sample size calculation, logistic regression analysis, and test–retest method.

Clinical studies have suggested that certain eating habits and dietary supplements may prevent or promote PCa. Our research did not find correlation between any specific dietary supplement and PCa, but multiple logistic regression analysis uncovered that BMI and total meat consumption was correlated with PCa. A growing body of literature has linked high intake of meat, particularly red meat and processed meat, to an increased risk of cancer. Evidence showed that this danger was not caused by meat per se, but rather by high-fat consumption or mutagenic heterocyclic amines produced by the ways of high-temperature cooking (grilled, barbequed, or deep frying) and processing procedures (smoking, seasoning or curing)^9,33^. Our data showed total meat consumption ≧ 1 serving per meal was at higher PCa risk than those < 1 serving per meal (OR = 2.74, 95% CI: 1.26–5.94, *p* = 0.011), but interestingly, the risk was not observed for red meat consumption. Methods of meat preparation were not investigated in this study. People who prefer high calorie intake of saturated animal meat and fat have often been associated with an increased risk of obesity and PCa, as subsequent increased testosterone levels are involved in prostate tissue proliferation^9^. Obesity may not only increase PCa incidence, but also increase risk of PCa mortality and recurrence^9,11,28^. Another mechanism is that obesity, especially when combined with physical inactivity, causes a decreased tissue response to insulin, notably in terms of glucose absorption. Insulin resistance causes chronically high blood insulin levels, which is a growth-promoting hormone and hence a scientifically reasonable risk factor for PCa. Obesity, deemed to have more metabolically active adipose tissue, is usually measured by higher BMI or larger waist circumference^11^. The BMI of subjects were also assessed in our study, noting higher BMI yielded slightly higher risk of PCa (OR = 1.15, 95% CI: 1.05–1.27, *p* = 0.003). Family history of PCa influences the risk of developing the disease^14^. Five single-nucleotide polymorphisms have been identified as having a statistically significant association with PCa when present in conjunction with a family history of PCa, according to Zheng et al.^12^. From 1986 to 2004, the Health Professional Follow-Up Study followed 3695 PCa patients and discovered increased PCa risk by 2.3 times, with a family history of PCa in both the father and a brother (95% CI: 1.76–3.12)^9^. Further evidence from twin studies elucidated that shared genetic factors were responsible for a large portion of familial aggregation of PCa, with a heritability estimate of 75%^11^. The data obtained from the forward stepwise logistic regression method in our study also revealed that those with a PCa family history had a higher risk of PCa (OR = 3.91, 95% CI: 1.17–13.12, *p* = 0.027). Sometimes, collinearity happens when different independent variables we use in a regression model are highly correlated with one another. Collinearity will inflate the variance and standard error of coefficient estimates. In other words, these independent variables with high correlation explain some of the same variance in the dependent variable, which reduces statistical significance. As a result, the model will become less reliable. One of the solutions to reduce or remove collinearity is to carefully select the variables or consider using other regression methods such as stepwise selection analysis. Collinearity may explain why p-value of PCa family history (0.027 and 0.074) was lower in forward stepwise selection analysis than in multiple logistic regression model.

All vegetarians were found in control group (8/135), while none were in PCa group (0/143), indicating that vegetarian lifestyle might have a protective effect for PCa (*p* = 0.003). Tantamango–Bartley et al.^34^ reported that vegan diets may confer a lower risk of PCa (HR = 0.65, 95% CI: 0.49–0.85), since vegans consumed a diet heavy of antioxidant-rich foods, reversing chronic inflammation induced by oxidative stress and free radical production that are known to damage genomic DNA and hasten PCa development. Another study from Taiwan conducted by Chen et al.^35^ recognized a beneficial association with vegan diets against PCa (OR = 0.67, 95% CI: 0.47–0.94).

While numerous studies worldwide have indicated the presence of HPV infection in PCa, we wanted to take a deeper look into Taiwan’s population. The study revealed the presence of HPV in two age-matching study groups, with case group comprising of PCa patients and control group comprising of BPH patients. Compared to the global average^36^, our study showed a lower HPV prevalence (7% to 18.93%) but a more notable OR (6.02 to 1.79). Singh et al.^23^ demonstrated HPV infection in 41% (39/95) of tumor biopsies and 20% (11/55) in BPH that served as disease controls, and a total of 92% HPV were type 16 and 18. Carozzi et al.^24^ found HPV DNA in 65.3% cancer and 48.0% benign biopsies without significance (*p* = 0.33), but high risk HPV type positivity in 53.8% (14/26) cancer and 20.0% (5/25) benign biopsies (*p* = 0.03), showing high risk types to be mainly 16, 18, 52, and 58. Leiros et al.^25^ detected HPV DNA in 41.5% (17/41) carcinoma samples, whereas all 30 hyperplasia samples were HPV-negative, but most HPV types were low risk or undefined. Serth et al.^26^ detected significantly higher copy numbers of HPV16-E6 sequences in the prostate tumors with 21% (10/47) when compared to the control tissue with 3% (1/37).

In our study, HPV DNA PCR amplification tested positive in 12 of total 278 patients (4.3%), of which 10 were amongst the 143 PCa patients (7%) and 2 amongst the 135 BPH patients (1.5%), with significant difference (OR = 6.02, 95% CI = 1.03–30.3, *p* = 0.046), suggesting association between HPV and PCa. However, CI of 95% was wide, and the difference observed was slightly significant. Additionally, if low risk types were not included, then high risk HPV detection would be deemed to be not associated with PCa. HPV frequencies in case group observed in the studies, from which sample size calculation was derived (Singh et al. 41%, Carozzi et al.53.8%, Leiros et al. 41.5%, and Serth et al. 21%), showed great discrepancy to that observed on our study (7%). Discrepancies in distribution and prevalence of HPV genotypes vary greatly across ethnic groups, geographic areas, and lifestyle groups, may also factor here. This is an observed limitation to our study, and future studies should re-calculate sample size for more robust results.

Sexual activity and STDs have been hypothesized to play a role in PCa development via a variety of pathophysiological routes, but the studies have thus far reported mixed results^13–17^. The multiple logistic regression in our study did not reveal significant difference in sexual behavior and STD history. Only multiple and lasting episodes of STDs could cause recurrent inflammation like chronic prostatitis and lead to escalating risk of PCa^37^. Although, in our study, 19.7% (28/142) of PCa patients had STD experience, which was higher than the 8.2% (11/135) in control group (*p* = 0.003), only a few had multiple lifelong STD episodes (1.4% amongst cases, and 3.0% amongst controls). Furthermore, almost all STD patients had sought prior medical treatment and achieved remission of the infection. These reasons may explain the insignificant results in the multivariate analysis.

Calculation of sample size, test–retest method, matching and adjustment for confounders are common techniques and an important part of study design in epidemiologic research. To our knowledge, these methods were not commonly used in prior case-control studies investigating correlation between HPV and PCa. Complete study design with these methods could be considered as strength of this study. Test–retest method is used to ensure the stability and reliability of survey scores across two time-points over a short period. If reliability is found lacking, there is little confidence that the data produced is an actual representation of the respondents. Assessment of personal or medical history is relatively problematic in elderly men due to age-related changes in performance, and we found the test–retest reliability crucial for our study. One drawback of the test–retest method is that it may be subject to recall bias, since respondents may just repeat what they previously claimed. Socially undesirable items like STDs or sexual behaviors are often under-reported, causing non-differential misclassification. In conclusion, our study uncovered additional protective factors for PCa, including regulating BMI by maintaining good physical activity and reducing total meat consumption. Middle-age and elderly men with PCa family history should stay highly vigilant about the occurrence of PCa. All these results could serve as a basis for designing PCa prevention strategies. It is suggested that clinicians educate patients on up-to-current findings in order to lower risk and promote effective prevention of PCa.

## Supplementary Information

Supplementary Information.

## Data Availability

All data generated or analysed during this study are included in this published article and its supplementary information files.
