# Author Correction: A matched case-control study in Taiwan to evaluate potential risk factors for prostate cancer

**DOI:** 10.1038/s41598-023-34567-0

**Published:** 2023-05-10

**Authors:** Heng-Jui Chang, Yuan-Hung Pong, Chen-Yen Chiang, Po-Chien Huang, Ming-Hua Wang, Yu-Jiun Chan, Tzuo-Yun Lan

**Affiliations:** 1 Institute of Public Health, National Yang Ming Chiao Tung University, Taipei, Taiwan; 2 Department of Radiation Oncology, Wesing Surgery Hospital, Kaohsiung, Taiwan; 3 Department of Urology, Ten Chen Hospital, Taoyuan, Taiwan; 4 Division of Urology, Department of Surgery, Taoyuan General Hospital, Ministry of Health and Welfare, Taoyuan, Taiwan; 5 Department of Urology, Min Sheng General Hospital, Taoyuan, Taiwan; 6 Department of Radiation Oncology, Landseed International Hospital, Taoyuan, Taiwan; 7 Division of Microbiology, Department of Pathology and Laboratory Medicine, Taipei Veterans General Hospital, Taipei, Taiwan; 8 Center for Infection Control, Taipei Veterans General Hospital, Taipei, Taiwan; 9 Institute of Hospital and Healthcare Administration, NationalYang Ming Chiao Tung University, No. 155, Sec. 2, Linong St., Beitou Dist., Taipei, 112304 Taiwan

Correction to: *Scientific Reports* https://doi.org/10.1038/s41598-023-31434-w, published online 16 March 2023

In the original version of this Article, Heng-Jui Chang was incorrectly listed as corresponding author. The correct corresponding author for this Article is Tzuo-Yun Lan. Correspondence and request for materials should be addressed to tylan@nycu.edu.tw.

The original Article has been corrected.

