# Supplementary material for: A matched case-control study in Taiwan to evaluate potential risk factors for prostate cancer
Source: Sci Rep. 2023 Mar 16;13:4382. doi: 10.1038/s41598-023-31434-w (PMC10020435; doi:10.1038/s41598-023-31434-w)
Supplement: Supplementary file 1 — Supplementary Information. [file 41598_2023_31434_MOESM1_ESM.docx]

Table S1 Comparison of Basic Demographic Characteristics

| Characteristic | Stratification | A-Case  (N=143) | | B-Control  (N=135) | | P value |
| --- | --- | --- | --- | --- | --- | --- |
|  |  | N | % | N | % |  |
| Age | 55 to 60 years | 6 | 4.2 | 11 | 8.1 | 0.826 |
|  | 60.1 to 65 years | 18 | 12.6 | 19 | 14.1 |  |
|  | 65.1 to 70 years | 35 | 24.5 | 30 | 22.2 |  |
|  | 70.1 to 75 years | 30 | 21.0 | 27 | 20.0 |  |
|  | 75.1 to 80 years | 28 | 19.6 | 25 | 18.5 |  |
|  | >80 years | 26 | 18.2 | 23 | 17.0 |  |
| Education | ≦High school | 110 | 76.9 | 101 | 74.8 | 0.681 |
|  | ≧College/Univ. | 33 | 23.1 | 34 | 25.2 |  |
| Marital status | Married | 117 | 81.8 | 114 | 84.4 | 0.559 |
|  | Others | 26 | 18.2 | 21 | 15.6 |  |
| Working status | FT/PT | 32 | 22.5 | 33 | 24.6 | 0.682 |
|  | RET/UE | 110 | 77.5 | 101 | 75.4 |  |
| Paternal hometown | Hoklo | 84 | 58.7 | 84 | 62.2 | 0.344 |
|  | Mainlander | 16 | 11.2 | 8 | 5.9 |  |
|  | Hakka | 40 | 28.0 | 42 | 31.1 |  |
|  | Aboriginal | 2 | 1.4 | 0 | 0 |  |
|  | Foreigner | 1 | 0.7 | 1 | 0.7 |  |
| Maternal hometown | Hoklo | 86 | 60.1 | 83 | 61.5 | 0.233 |
|  | Mainlander | 14 | 9.8 | 8 | 5.9 |  |
|  | Hakka | 40 | 28.0 | 43 | 31.9 |  |
|  | Aboriginal | 3 | 2.1 | 0 | 0 |  |
|  | Foreigner | 0 | 0 | 1 | 0.7 |  |

Univ. = university; FT = full-time; PT = part-time; RET = retired; UE = unemployed; *P<0.05.

Table S2 Comparison of Radiation Therapy History, Dietary Supplement and Drug History

| Characteristic | Stratification | A-Case  (N=143) | | B-Control  (N=135) | | P value |
| --- | --- | --- | --- | --- | --- | --- |
|  |  | n | % | n | % |  |
| Radiation therapy | No | 138 | 97.2 | 131 | 97.0 | 1.000 |
| history | Yes | 4 | 2.8 | 4 | 3.0 |  |
| Dietary supplement |  |  |  |  |  |  |
| Calcium | No | 124 | 86.7 | 115 | 85.2 | 0.714 |
|  | Yes | 19 | 12.3 | 20 | 14.8 |  |
| Vitamin B | No | 130 | 90.9 | 123 | 91.1 | 0.953 |
|  | Yes | 13 | 9.1 | 12 | 8.9 |  |
| Vitamin C | No | 138 | 96.5 | 128 | 94.8 | 0.489 |
|  | Yes | 5 | 3.5 | 7 | 5.2 |  |
| Vitamin complex | No | 124 | 86.7 | 110 | 81.5 | 0.232 |
|  | Yes | 19 | 13.3 | 25 | 18.5 |  |
| Zinc | No | 142 | 99.3 | 133 | 98.5 | 0.613 |
|  | Yes | 1 | 0.7 | 2 | 1.5 |  |
| Selenium | No | 143 | 100 | 135 | 100 | NA |
|  | Yes | 0 | 0 | 0 | 0 |  |
| Green tea powder | No | 142 | 99.3 | 135 | 100 | 1.000 |
|  | Yes | 1 | 0.7 | 0 | 0 |  |
| Isoflavone | No | 141 | 98.6 | 134 | 99.3 | 1.000 |
|  | Yes | 2 | 1.4 | 1 | 0.7 |  |
| Royal jelly | No | 143 | 100 | 134 | 99.3 | 0.486 |
|  | Yes | 0 | 0 | 1 | 0.7 |  |
| Phytoestrogen | No | 143 | 100 | 135 | 100 | NA |
| essential | Yes | 0 | 0 | 0 | 0 |  |
| Other supplements | No | 119 | 83.2 | 104 | 77.0 | 0.196 |
|  | Yes | 24 | 16.8 | 31 | 23.0 |  |
| Drug history |  |  |  |  |  |  |
| NSAID | No | 128 | 89.5 | 124 | 91.9 | 0.503 |
|  | Yes | 15 | 10.5 | 11 | 8.1 |  |
| Anti-hypertensive | No | 73 | 51.0 | 70 | 51.9 | 0.894 |
|  | Yes | 70 | 49.0 | 65 | 48.1 |  |
| Hypolipidemic agent | No | 115 | 80.4 | 104 | 77.0 | 0.491 |
|  | Yes | 28 | 19.6 | 31 | 23.0 |  |
| Anti-alopecia agent | No | 143 | 100 | 134 | 99.3 | 0.486 |
|  | Yes | 0 | 0 | 1 | 0.7 |  |
| Androgen hormone | No | 143 | 100 | 135 | 100 | NA |
|  | Yes | 0 | 0 | 0 | 0 |  |
| Others | No | 108 | 75.5 | 101 | 74.8 | 0.891 |
|  | Yes | 35 | 24.5 | 34 | 25.2 |  |

NSAID = non-steroid anti-inflammatory drug; NA = not assessed; *P<0.05

Table S3 History of Pelvic Disease and Specific Status Related with Chronic Inflammation

| Characteristic | Stratification | A-Case  (N=143) | | B-Control  (N=135) | | P value | |
| --- | --- | --- | --- | --- | --- | --- | --- |
|  |  | n | % | n | % |  |  |
| Urinary calculi | No | 101 | 70.6 | 99 | 73.3 | 0.616 |  |
|  | Yes | 42 | 29.4 | 36 | 26.3 |  |  |
| Urinary tract infection | No | 101 | 70.6 | 105 | 77.8 | 0.174 |  |
|  | Yes | 42 | 29.4 | 30 | 22.2 |  |  |
| Bladder disease | No | 137 | 95.8 | 130 | 96.3 | 0.833 |  |
|  | Yes | 6 | 4.2 | 5 | 3.7 |  |  |
| Pelvic trauma | No | 136 | 95.1 | 129 | 95.6 | 0.859 |  |
|  | Yes | 7 | 4.9 | 6 | 4.4 |  |  |
| Pelvic surgery | No | 121 | 84.6 | 112 | 83.0 | 0.709 |  |
|  | Yes | 22 | 15.4 | 23 | 17.0 |  |  |
| Autoimmune disease | No | 143 | 100 | 134 | 99.3 | 0.486 |  |
|  | Yes | 0 | 0 | 1 | 0.7 |  |  |
| Pelvic cancer | No | 139 | 97.2 | 129 | 95.6 | 0.531 |  |
|  | Yes | 4 | 2.8 | 6 | 4.4 |  |  |
| Extrapelvic cancer | No | 141 | 99.3 | 129 | 95.6 | 0.061 |  |
|  | Yes | 1 | 0.7 | 6 | 4.4 |  |  |

*P<0.05

Table S4 Personal Lifestyles

| Characteristic | Stratification | A-Case  (N=143) | | B-Control  (N=135) | | P value |
| --- | --- | --- | --- | --- | --- | --- |
|  |  | n | % | n | % |  |
| Dietary habit |  |  |  |  |  |  |
| Tea drinking | No | 53 | 40.2 | 44 | 32.6 | 0.315 |
| habit | 1-4 times per week | 50 | 37.9 | 52 | 38.5 |  |
|  | Almost everyday | 29 | 22.0 | 39 | 28.9 |  |
| Exercise | No | 39 | 29.5 | 36 | 26.7 | 0.732 |
|  | Occasional | 43 | 32.6 | 50 | 37.0 |  |
|  | Everyday | 50 | 37.9 | 49 | 36.3 |  |
| Smoking | Never | 55 | 41.7 | 58 | 43.0 | 0.830 |
|  | Current/former | 77 | 58.3 | 77 | 57.0 |  |
| Betel nut chewing | Never | 101 | 76.5 | 112 | 83.6 | 0.173 |
|  | Former | 29 | 22.0 | 22 | 16.4 |  |
|  | Current | 2 | 1.5 | 0 | 0 |  |
| Alcohol drinking | Never | 53 | 40.2 | 70 | 51.9 | 0.157 |
|  | Abstainer | 18 | 13.6 | 14 | 10.4 |  |
|  | Current | 61 | 46.2 | 51 | 37.8 |  |

*P<0.05

Table S5 Family History of PCa

| Characteristic | Stratification | A-Case  (N=143) | | B-Control  (N=135) | | P value |
| --- | --- | --- | --- | --- | --- | --- |
|  |  | n | % | n | % |  |
| Family member with PCa |  |  |  |  |  |  |
| Grandfather | No | 135 | 98.5 | 130 | 100 | 0.498 |
|  | Yes | 2 | 1.5 | 0 | 0 |  |
| Granduncle | No | 133 | 100 | 130 | 100 | NA |
|  | Yes | 0 | 0 | 0 | 0 |  |
| Father | No | 133 | 97.1 | 128 | 98.5 | 0.685 |
|  | Yes | 4 | 2.9 | 2 | 1.5 |  |
| Uncle | No | 82 | 97.6 | 81 | 100 | 0.497 |
|  | Yes | 2 | 2.4 | 0 | 0 |  |
| Brother | No | 131 | 94.9 | 126 | 96.9 | 0.411 |
|  | Yes | 7 | 5.1 | 4 | 3.1 |  |
| Cousin | No | 134 | 100 | 129 | 99.2 | 0.492 |
|  | Yes | 0 | 0 | 1 | 0.8 |  |
| Son | No | 138 | 100 | 130 | 100 | NA |
|  | Yes | 0 | 0 | 0 | 0 |  |
| Grandson | No | 134 | 100 | 130 | 100 | NA |
|  | Yes | 0 | 0 | 0 | 0 |  |

*P<0.05

Table S6 History of Androgen Related Therapy and Reproductive System

| Characteristic | Stratification | A-Case  (N=143) | | B-Control  (N=135) | | P value |
| --- | --- | --- | --- | --- | --- | --- |
|  |  | n | % | n | % |  |
| Androgen replacement | Never | 136 | 97.1 | 129 | 95.6 | 0.535 |
| therapy | Ever | 4 | 2.9 | 6 | 4.4 |  |
| Anti-alopecia agent | Never | 134 | 100 | 131 | 99.2 | 0.496 |
|  | Ever | 0 | 0 | 1 | 0.8 |  |
| Vasectomy | Never | 135 | 96.4 | 125 | 92.6 | 0.161 |
|  | Ever | 5 | 3.6 | 10 | 7.4 |  |
|  | Ever | 28 | 20.0 | 13 | 9.6 |  |
| Genital beading | Never | 139 | 100 | 133 | 99.3 | 0.491 |
|  | Ever | 0 | 0 | 1 | 0.7 |  |
| Tattoo at lower abdomen or | Never | 139 | 99.3 | 135 | 100 | 1.000 |
| genital organ | Ever | 1 | 0.7 | 0 | 0 |  |
| HPV vaccination | No | 139 | 99.3 | 135 | 100 | 1.000 |
|  | Yes | 1 | 0.7 | 0 | 0 |  |
| IIEF-5 (mean±sd) |  | 8.1±9.5 | | 8.3±9.5 | | 0.842 |

*P<0.05

Table S7 Sexual Behavior and STD

| Characteristic | Stratification | A-Case  (N=143) | | B-Control  (N=135) | | P value |
| --- | --- | --- | --- | --- | --- | --- |
|  |  | n | % | n | % |  |
| Sexual orientation | Heterosexual | 139 | 97.0 | 134 | 99.3 | 0.372 |
|  | Homosexual | 4 | 2.8 | 1 | 0.7 |  |
| Marriage | ≦Once | 130 | 90.9 | 126 | 93.3 | 0.454 |
|  | ≧Twice | 13 | 9.1 | 9 | 6.7 |  |
| Children | ≦1 child | 15 | 10.5 | 17 | 12.6 | 0.583 |
|  | ≧2 children | 128 | 89.5 | 118 | 87.4 |  |
| Sexual partner in recent one year | None | 81 | 57.0 | 74 | 54.8 | 0.709 |
|  | ≧1 person | 61 | 43.0 | 61 | 45.2 |  |
| Years using condoms | Never/unwilling to tell | 80 | 56.3 | 59 | 43.7 | 0.094 |
|  | <5 years | 42 | 29.6 | 48 | 35.6 |  |
|  | ≧5years | 20 | 14.1 | 28 | 20.7 |  |
| Prostitution | Never/unwilling to tell | 75 | 52.4 | 88 | 65.2 | 0.057 |
|  | 1-2 persons | 21 | 14.7 | 19 | 14.1 |  |
|  | ≧3 persons | 47 | 32.9 | 28 | 20.7 |  |

*P<0.05

Table S8 Sexual Behavior and Reproductive Status in Spouse

| Characteristic | Stratification | A-Case  (N=143) | | B-Control  (N=135) | | P value |
| --- | --- | --- | --- | --- | --- | --- |
|  |  | N | % | n | % |  |
| STD history | Never/unwilling to tell | 142 | 99.3 | 134 | 99.3 | 1.000 |
|  | Ever | 1 | 0.7 | 1 | 0.7 |  |
| Cancer history | No | 131 | 91.6 | 121 | 89.6 | 0.904 |
|  | GYN or pelvic cancer | 4 | 2.8 | 6 | 4.4 |  |
|  | HN/RESP/GI cancer | 3 | 2.1 | 3 | 2.2 |  |
|  | Others | 5 | 3.5 | 5 | 3.7 |  |
| HPV vaccination | No/unknown | 143 | 100 | 133 | 98.5 | 0.235 |
|  | Yes | 0 | 0 | 2 | 1.5 |  |

GYN = gynecologic; HN = head and neck; RESP = respiratory tract; GI = gastrointestinal; *P<0.05

Table S9 Assessing Test-retest Reliability of the Questionnaires

| Phase | Question number | Total (n=40) | Case (n=20) | Control (n=20) |
| --- | --- | --- | --- | --- |
|  |  | CC | CC | CC |
| Basic data | Q1-1 | 1.000 | 1.000 | 1.000 |
|  | Q1-2 | 1.000 | 1.000 | 1.000 |
|  | Q1-3 | 1.000 | 1.000 | 1.000 |
|  | Q1-4 | 1.000 | 1.000 | 1.000 |
|  | Q1-5 | 1.000 | 1.000 | 1.000 |
|  | Q1-6 (father) | 1.000 | 1.000 | 1.000 |
|  | Q1-6 (mother) | 1.000 | 1.000 | 1.000 |
|  | Q1-7 (height) | 1.000 | 1.000 | 1.000 |
|  | Q1-7 (weight) | 0.999 | 1.000 | 0.999 |
|  | Q1-8 | 1.000 | 1.000 | 1.000 |
| Dietary habit | Q2-1 | 0.863 | 0.842 | 0.943 |
|  | Q2-2 | 0.940 | 1.000 | 0.892 |
|  | Q2-3 | 0.907 | 0.791 | 1.000 |
|  | Q2-4 | 0.897 | 1.000 | 0.784 |
|  | Q2-5 (veg） | 0.971 | 1.000 | 0.932 |
|  | Q2-5 (fruit) | 0.894 | 1.000 | 0.800 |
|  | Q2-5 (soybean) | 0.896 | 0.905 | 0.891 |
|  | Q2-6 | 1.000 | 1.000 | 1.000 |
|  | Q2-7 | 0.945 | 0.809 | 0.966 |
|  | Q2-8 | 0.899 | 0.808 | 0.980 |
|  | Q2-9 | 0.944 | 0.895 | 1.000 |
|  | Q2-10 | 1.000 | 1.000 | 1.000 |
|  | Q2-11 | 0.981 | 1.000 | 0.971 |
|  | Q2-12 | 0.936 | 1.000 | 0.889 |
|  | Q2-13 | 1.000 | 1.000 | 1.000 |
|  | Q2-14 | 1.000 | 1.000 | 1.000 |
| Family prostate | Q3-1 | 1.000 | 1.000 | 1.000 |
| disease | Q3-2 | 1.000 | 1.000 | 1.000 |
|  | Q3-3 | 1.000 | 1.000 | 1.000 |
|  | Q3-4 | 1.000 | 1.000 | 1.000 |
|  | Q3-5 | 1.000 | 1.000 | 1.000 |
|  | Q3-6 | 1.000 | 1.000 | 1.000 |

CC = correlation coefficient

Pearson’s r≧0.7 indicates acceptable reliability.

Table S9 Assessing Test-retest Reliability of the Questionnaires (Continued)

| Phase | Question number | | Total(n=40) | | | Case (n=20) | | Control (n=20) |
| --- | --- | --- | --- | --- | --- | --- | --- | --- |
|  |  |  | CC | | | CC | | CC |
| Androgen related | Q4-1 | | 1.000 | | 1.000 | | | 1.000 |
| therapy | Q4-2 | | - | | - | | | - |
|  | Q4-3 | | - | | - | | | - |
|  | Q4-4 | | - | | - | | | - |
|  | Q4-5 | | 1.000 | | 1.000 | | | 1.000 |
|  | Q4-6 | | - | | - | | | - |
|  | Q4-7 | | - | | - | | | - |
|  | Q4-8 | | - | | - | | | - |
|  | Q4-9 | | - | | - | | | - |
| Reproductive | Q5-1 | | 1.000 | | 1.000 | | | 1.000 |
| system | Q5-2 | | 1.000 | | 1.000 | | | 1.000 |
|  | Q5-3 | | 1.000 | | 1.000 | | | 1.000 |
|  | Q5-4 | | 1.000 | | 1.000 | | | 1.000 |
|  | Q5-5 | | 1.000 | | 1.000 | | | 1.000 |
|  | IPSS | | 0.858 | | 0.932 | | | 0.800 |
|  | IIEF-5 | | 0.966 | | 0.992 | | | 1.000 |
| Sexual behavior | Q6-1 | | 1.000 | | 1.000 | | | 1.000 |
| and STD | Q6-2 | | 1.000 | | 1.000 | | | 1.000 |
|  | Q6-3 | | 1.000 | | 1.000 | | | 1.000 |
|  | Q6-4 | | 0.981 | | 0.968 | | | 0.994 |
|  | Q6-5 | | 0.725 | | 0.935 | | | 0.634 |
|  | Q6-6 | | 0.955 | | 1.000 | | | 0.904 |
|  | Q6-7 | | 0.995 | | 0.989 | | | 1.000 |
|  | Q6-8 | | 1.000 | | 1.000 | | | 1.000 |
|  | Q6-9 | | 1.000 | | 1.000 | | | 1.000 |
| Sexual behavior | Q7-1 | | | 0.850 | | | 0.783 | 1.000 |
| and reproductive | Q7-2 | | | 1.000 | | | 1.000 | 1.000 |
| system in spouse | Q7-3 | | | 1.000 | | | 1.000 | 1.000 |
|  | Q7-4 | | | 1.000 | | | 1.000 | 1.000 |
|  | Q7-5 | 0.780 | | | 0.833 | | | 0.653 |

CC = correlation coefficient

Pearson’s r≧0.7 indicates acceptable reliability.
